# Separation of Biological Entities from Human Blood by Using Magnetic Nanocomposites Obtained from Zeolite Precursors

**DOI:** 10.3390/molecules25081803

**Published:** 2020-04-14

**Authors:** Serena Esposito, Antonello Marocco, Gianfranco Dell’Agli, Barbara Bonelli, Franca Mannu, Paolo Allia, Paola Tiberto, Gabriele Barrera, Michele Pansini

**Affiliations:** 1 Department of Applied Science and Technology and INSTM Unit of Torino-Politecnico, Corso Duca degli Abruzzi 24, Politecnico di Torino, 10129 Torino, Italy; serena_esposito@polito.it (S.E.); paolo.allia@formerfaculty.polito.it (P.A.); 2 Department of Civil and Mechanical Engineering and INSTM Research Unit, Università degli Studi di Cassino e del Lazio Meridionale, Via G. Di Biasio 43, 03043 Cassino, FR, Italy; a.marocco@unicas.it (A.M.); dellagli@unicas.it (G.D.); 3 PoliTO BioMED Interdepartmental LAB, Corso Duca degli Abruzzi 24, 10129 Torino, Italy; 4 Nurex srl, Z.I. Predda Niedda, 07100 Sassari, Italy; franca.mannu@nurex.it; 5 INRIM, Nanoscience and Materials Division, Strada delle Cacce 91, 10135 Torino, Italy; p.tiberto@inrim.it (P.T.); g.barrera@inrim.it (G.B.)

**Keywords:** biological separations, porous metal–ceramic nanocomposites, magnetic nanoparticles, zeolite precursor, thermal treatment, adsorption

## Abstract

In this work, three novel magnetic metal–ceramic nanocomposites were obtained by thermally treating Fe-exchanged zeolites (either A or X) under reducing atmosphere at relatively mild temperatures (750–800 °C). The so-obtained materials were thoroughly characterized from the point of view of their physico-chemical properties and, then, used as magnetic adsorbents in the separation of the target gene factors V and RNASE and of the *Staphylococcus aureus* bacteria DNA from human blood. Such results were compared with those obtained by using a top ranking commercial separation system (namely, SiMAG-N-DNA by Chemicell). The results obtained by using the novel magnetic adsorbents were similar to (or even better than) those obtained by using the commercial system, both during manual and automated separations, provided that a proper protocol was adopted. Particularly, the novel magnetic adsorbents showed high sensitivity during tests performed with small volumes of blood. Finally, the feasible production of such magnetic adsorbents by an industrial process was envisaged as well.

## 1. Introduction

Separation of biological entities from biological fluids is the mandatory prerequisite of many biochemical and diagnostic processes [1]. Several methods for biological entity purification have been developed, so far, spanning from traditional fluid-phase methods, based on a complex series of precipitation and washing steps, to alternative solid-phase techniques, based on either ionic exchange in aqueous environment (by means of an anion exchanger) or adsorption on hydrophilic matrices (typically silica) under chaotropic conditions [2,3,4,5,6,7,8,9,10]. The scientific and technologic community is paying increasing attention to the use of magnetic solid adsorbents that can easily be separated from biological fluids by using an external magnet, the adsorbed species being, then, recovered by a simple elution step [11,12]. 

The most common magnetic materials/systems used in biological separations imply the use of nanoparticles with an iron oxide core (either magnetite (Fe_3_O_4_) or maghemite (γ-Fe_2_O_3_), usually) covered by a porous silica shell; the core is responsible for the magnetic behavior, while the surface shell adsorbs some targeted biological entities [11,12,13,14], as described by a great deal of literature reports on their preparation and characterization [1,11,12,13,14,15,16,17,18,19,20,21,22,23,24]. However, the use of such systems in the separation of nucleic acids from biological fluids does not appear free from troubles. In particular, the proper manufacture of their complicated microstructures is usually attained by laborious, multi-step, and lengthy techniques (e.g., sol–gel assisted co-precipitation), which require both skillful chemists and expensive reactants. As further drawbacks, only a few grams of final product can be obtained in a single batch; some syntheses are poorly reproducible (giving rise to slightly different magnetic adsorbents that, in turn, require different settings of the used apparatus) and the final product is expensive [20,21,22]. All the aforementioned aspects are seen as crucial issues, especially by the insiders.

Such considerations explain the growing demand of alternative techniques of production of magnetic carriers for biological separations. To this end, a patented process, set up by some of us [25,26], could be used in the manufacture of reliable magnetic adsorbents. In such a process, the employed raw materials are commercial zeolites (price per kg, some dozens of Euros), the use of which is widespread in many technological sectors [27,28]. In the process [25,26], zeolites are repeatedly exchanged with transition metal ions (here, Fe^2+^) and then subjected to chemical reduction by a thermal treatment at relatively mild temperature (600–850 °C) under reducing atmosphere. Such thermal treatment gives rise to the reduction of a fraction of the exchanged iron ions to Fe^0^ species and to the simultaneous structural collapse of the parent zeolite; the final product is a metal–ceramic nanocomposite, where nanoparticles of Fe^0^ are dispersed within a ceramic matrix, which, in turn, still exhibits some irregular porosity, reminiscent of the parent zeolite matrix, and which mostly consists of amorphous silica and alumina, along with other Fe-containing phases, i.e., FeO_x_ and Fe silicates, vide infra [29,30,31,32,33,34]. The same metal–ceramic nanocomposites, which have magnetic properties [29,30,31,32,33,34], were already successfully used for a broad range of applications, i.e., in the removal of agrochemicals from water by adsorption [35,36], as simulants of lunar agglutinates [37], and in the separation of *Escherichia coli* DNA from a crude cell lysate [38]. Indeed, the results obtained in [38] appeared particularly interesting for biomolecule separation, since the produced adsorbents were able to separate *Escherichia coli* DNA with a yield and an extraction efficiency that were about two or three times higher than those obtained by using a commercial system (GenElute Bacterial Genomic DNA kit from Sigma-Aldrich). 

The above considerations and results triggered the present study, in which some of the metal–ceramic nanocomposites produced according to [29,30,31,32,33,34,35,36,37,38] were tested for some biological separations that are usually performed in molecular diagnostic laboratories. In particular, the produced magnetic adsorbents were used here for the separation of the target gene factors V and RNASE and of the *Staphylococcus aureus* bacterium from human blood samples. Indeed, *Staphylococcus aureus* is one of the most common and lethal causes of bloodstream infection, sepsis, and sepsis shock, with an incidence that is increasing also in Europe and in the US; worldwide, sepsis is one of the main causes of urgent hospitalization and of mortality. For instance, an observational study carried out in a Norwegian county [39] showed that *Staphylococcus aureus* bloodstream infection brought a high case fatality rate, especially among patients with severe sepsis and septic shock and among those with pulmonary or an unknown focus of infection. During the studied period (1996–2011), no decrease in 30- or 90-day mortality risk was observed, showing the need for continuous surveillance and of further diagnostic and therapeutic efforts in order to fight such a serious disease. The availability of rapid and affordable detection methods [40] of bacteria and of their metabolites also affects the therapeutic efficiency. Indeed, beyond clinical parameters, sepsis is a serious disease for which the diagnosis is usually carried out by searching the pathogen by culture method (currently, the gold standard method), which responds only after 24–72 h or even longer time. The culture method is the gold standard only when it is positive, whereas the method gives a negative response with patients under antibiotic therapy, since bacterial growth is inhibited. Instead, diagnosis can be obtained within hours of sampling by real-time PCR (polymerase chain reaction), a quantitative technique for rapid microbiological diagnosis. This means the possibility of starting quickly a targeted antibiotic therapy for the found microorganism(s). Furthermore, real-time PCR sensitivity is fundamental from the diagnostic point of view; the higher the sensitivity, the higher the ability to “catch” low concentrations of pathogens in blood.

In this work, the same separations were also performed by using one of the best performing commercial separation systems, i.e., SiMAG-N-DNA produced by Chemicell, hereafter referred to as Chemicell NPs (N-DNA Particles). The latter is a magnetic fluid/ferrofluid, i.e., an aqueous colloidal dispersion of superparamagnetic particles with a silica shell and a maghemite core, able to guarantee a sufficient response to the applied magnetic field, simultaneously having almost zero remanence, an important parameter in magnetic separations, because the lower the remanence, the better the particle dispersibility after switching off the external magnetic field [20]. Such magnetic properties allow use of the system in automated separations, for instance. 

The whole set of the reported experiments allowed evaluation of the performance of the proposed adsorbents for the separation of biological entities as well as comparison of their performance to that of the top ranking commercial system.

## 2. Results and Discussion

### 2.1. Physico-Chemical Characterization of the Magnetic Adsorbents

Table 1 reports some details about preparation of the three magnetic adsorbents that are object of this study.

According to chemical analysis, the Fe-A zeolite contained 4.51 meq g^−1^ Fe^2+^ and 0.58 meq g^−1^ Na^+^, and the Fe-X zeolite contained 3.54 meq g^−1^ Fe^2+^ and 1.15 meq g^−1^ Na^+^. The calculated cation equivalent fractions (as calculated by using the chemical analysis data) were x_Fe_ = 0.89 and x_Na_ = 0.11 (Fe-A), and x_Fe_ = 0.75 and x_Na_ = 0.25 (Fe-X). The iron content of the two Fe-exchanged zeolites was used to calculate the wt.% Fe in the final sample, which, by assuming fully dehydrated materials, were 16.9 wt.% for both FeA750C2h and FeA800C0min magnetic adsorbents and 13.1 wt.% for FeX750C2h. Seemingly, the wt.% Fe were correlated to the (lower) cation exchange capacity of Na-X zeolite with respect to Na-A and, accordingly, the parent zeolites were subjected to a different number of exchanges, as reported in the Materials and Methods section. 

**Figure 1:**
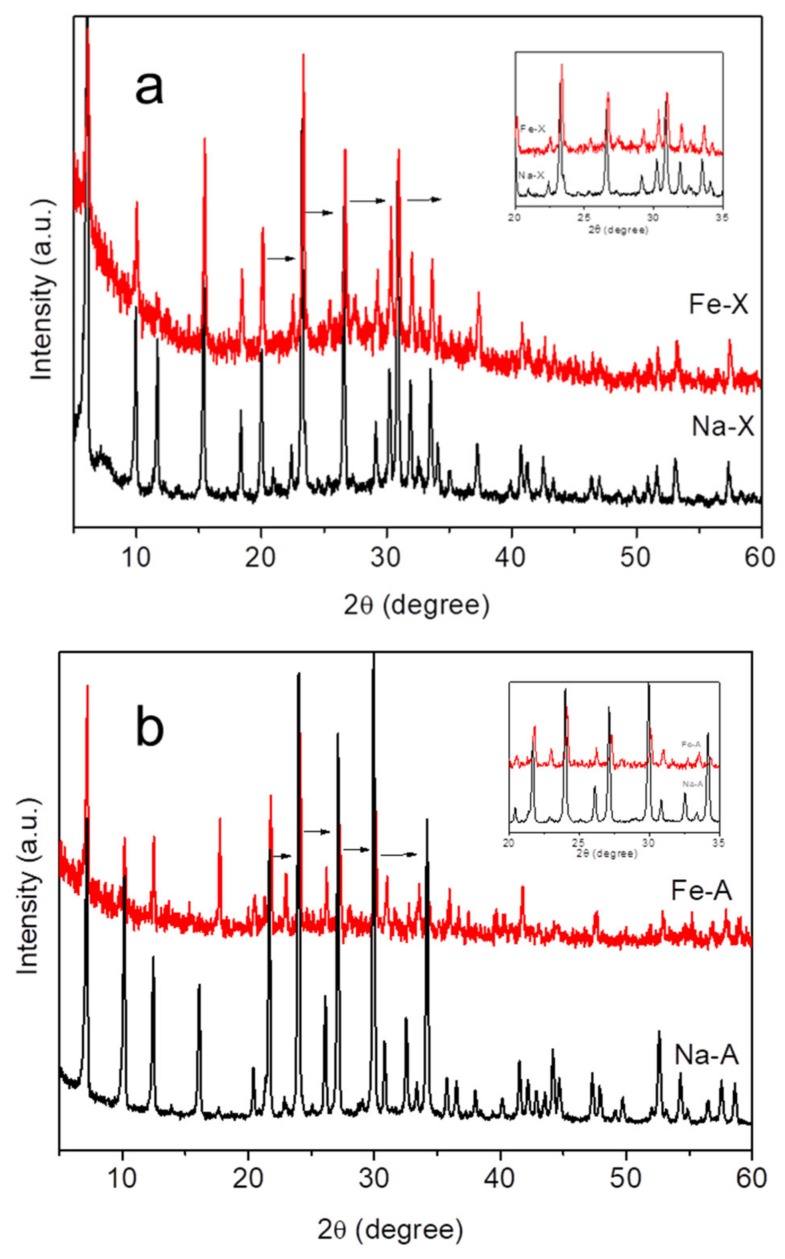
Section (**a**): powder XRD patterns of Na-X and Fe-X zeolites; section (**b**): powder XRD patterns of Na-A and Fe-A zeolites.

Figure 1 reports the XRD patterns of the Na-X, Fe-X (a) and Na-A, Fe-A (b) zeolites. Some slight differences between the XRD patterns of the Na- and Fe-zeolites could be noticed, as seen in both Figure 1a,b. Particularly, after cation exchange i) some peaks (inset) appeared slightly shifted (arrows); ii) most of the peaks were reduced in intensity (red curves); and iii) some minor peaks exhibited higher intensity. Such findings can be reasonably explained by small changes of the zeolite unit cell volume arising from Fe-exchange and/or the larger X-ray absorption coefficient of Fe^2+^ ions, as previously found with Ba^2+^ exchanged A zeolite [41]. 

**Figure 2:**
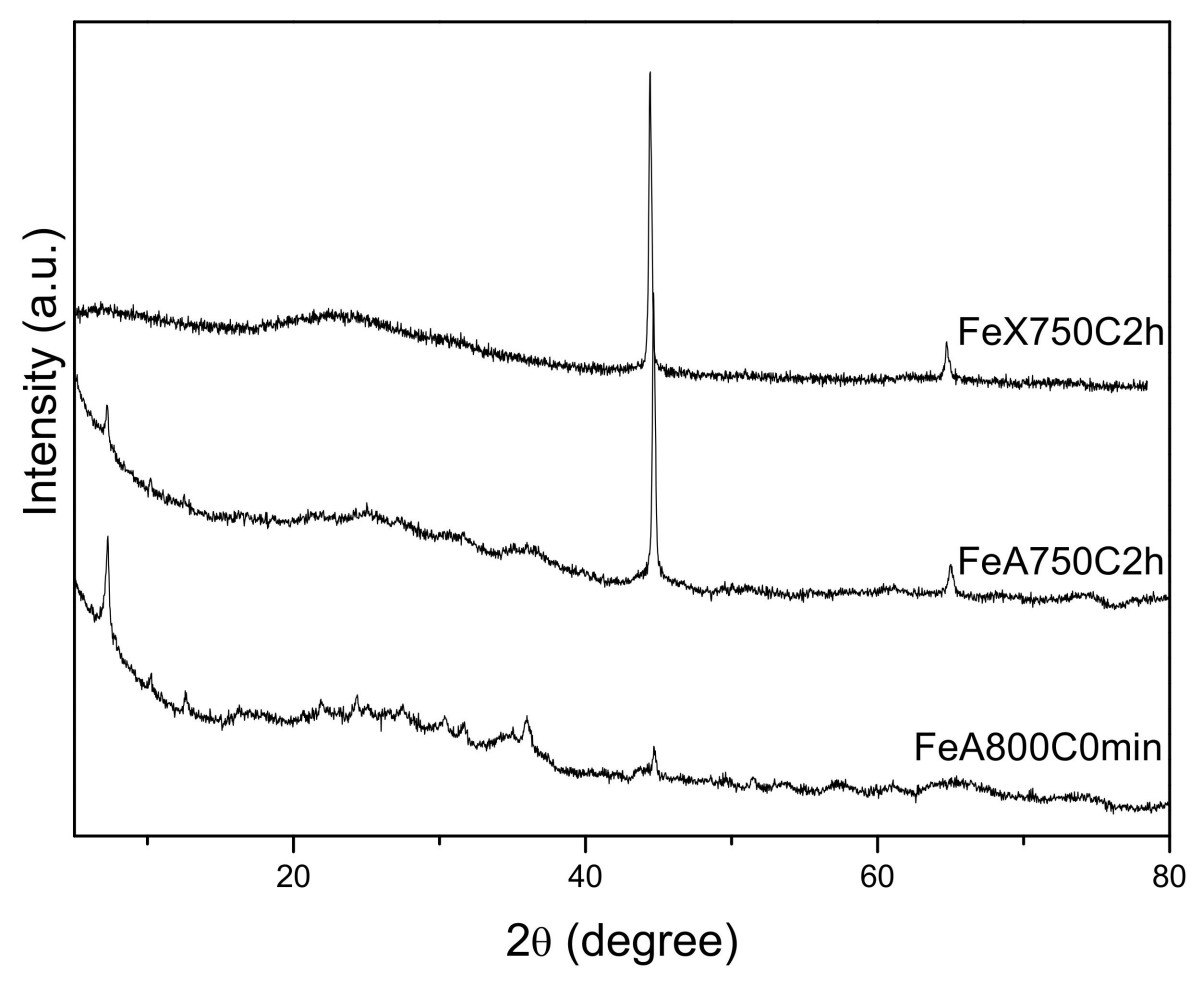
From bottom to top: powder XRD patterns of FeA800C0min, FeA750C2h, and FeX750C2h samples.

Figure 2 reports the powder XRD patterns of the studied magnetic adsorbents. Reduction of Fe^2+^ cations by H_2_, which leads to H_2_O production by consumption of framework oxygen, appeared evident in the XRD patterns of both FeA750C2h and FeX750C2h and, to a minor extent, in that of FeA800C0min (vide infra). Indeed, a sharp peak at 44.6 2*ϴ* and a broad signal centered at about 24 2*ϴ* were observed with the first two patterns. The sharp peak was ascribed to the most intense diffraction peak of metallic iron (Fe^0^ species), arising from the reduction of Fe^2+^ cations by H_2_ and less abundant with the FeA800C0min sample, which underwent a very short thermal treatment, and the broad signal to the occurrence of an amorphous phase related to the zeolite structure collapse. A small amount of residual zeolite and of fayalite (Fe_2_SiO_4_) were observed in the XRD patterns of FeA750C2h and FeA800C0min, but not in that of FeX750C2h. 

Such XRD results were corroborated by the Rietveld quantitative phase analysis (QPA) results reported in Table 2. The most relevant result was the amount of (reduced) Fe^0^, corresponding to 2.6 wt.% Fe (FeA750C2h), 0.3 wt.% Fe (FeA800C0min), and 1.6 wt.% Fe (FeX750C2h). Apparently, the rest of the iron was contained in the amorphous phase (89.4, 85.8, and 98.4 (wt.%), respectively) resulting from zeolite structure thermal collapse. Moreover, the presence of small amounts of parent zeolite (1.0 wt.% and 2.4 wt.%) and of fayalite (Fe_2_SiO_4_) in both FeA750C2h (7.0 wt.%) and FeA800C0min (11.5 wt.%) explained the corresponding smaller amount of amorphous phase occurring in zeolite A-derived adsorbents, as compared to the amount of amorphous phase occurring in FeX750C2h. The larger amount of Fe^0^ in sample FeA750C2h than in sample FeX750C2h was likely related to a more expedited iron reduction in the A zeolite structure (rather than in X zeolite), in agreement with the literature [31], whereas the low Fe^0^ content in the FeA800C0min sample (only 0.3 wt.%) may be ascribed to the extremely short reduction time (i.e., 0 min) adopted for its thermal reduction.

The values of specific surface area (S_BET_), total pore volume (V_p_), and micropore volume (V_mp_) of the FeA750C2h and FeX750C2h magnetic adsorbents (Table 2) were considerably lower than those of the parent zeolites, confirming zeolite structure thermal collapse followed by a process of coalescence, which increased the overall compactness of the final products. Conversely, the FeA800C0min magnetic adsorbent exhibited a high value of specific surface area (S_BET_). Seemingly, the shorter thermal treatment resulted not only in a lower amount of reduced iron species (Fe^0^), but also in a lower extent of structural collapse and coalescence, leading to a material with larger specific surface area (and porosity).

**Figure 3:**
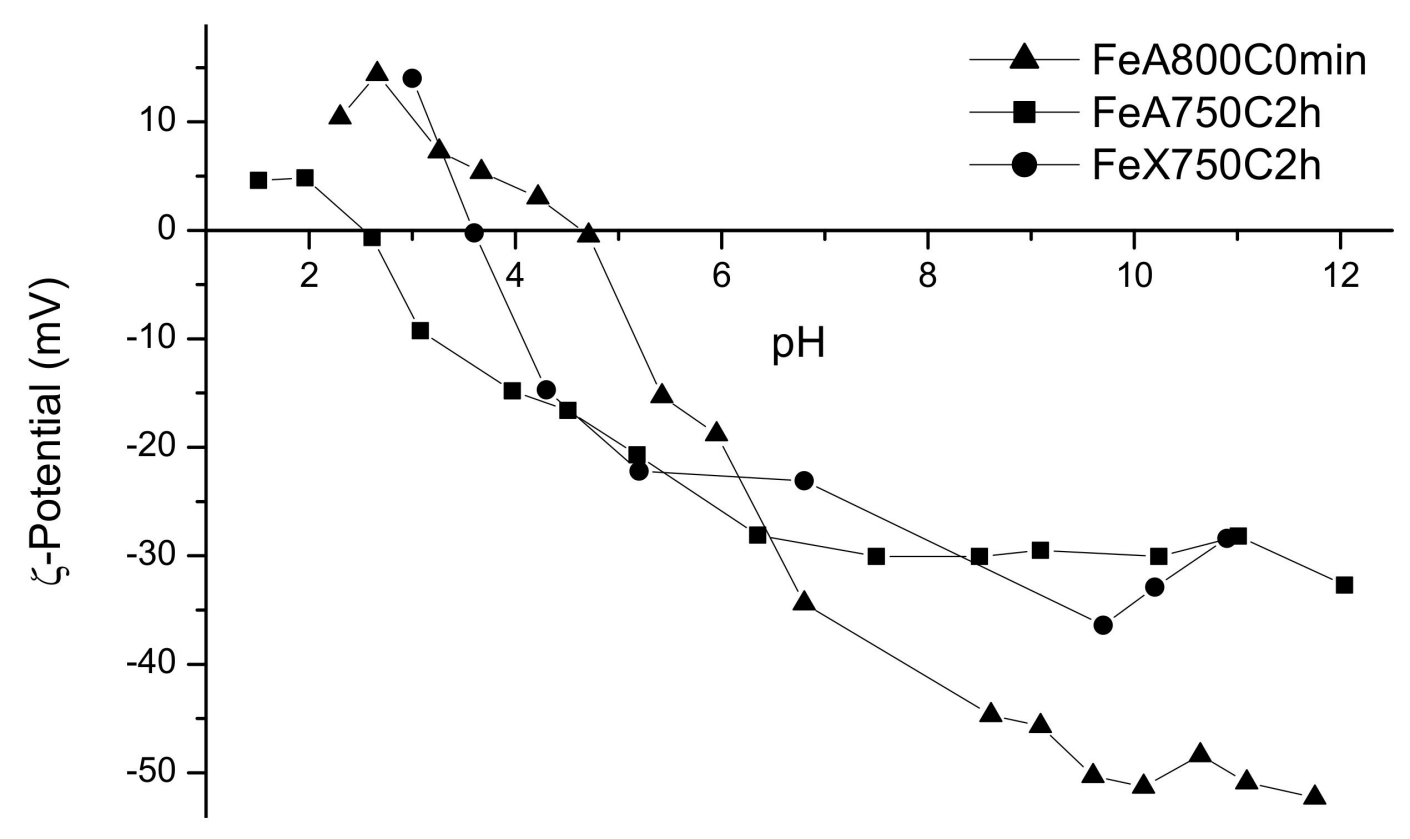
ζ-potential curves of FeA750C2h (squares), FeA800C0min (triangles), and FeX750C2h (circles).

For adsorbents in the liquid phase, the surface area and porosity were not the only important parameters, as solids have a surface charge in water, which varies with pH; therefore, the ζ-potential curves of the three magnetic adsorbents are reported in Figure 3. Interestingly, the pH of zero charge of the FeA7502h and FeA800C0min was ca. 2.6 and 4.7, respectively, in agreement with the fact that the thermal treatment had a deep effect also on the surface properties of the materials, which were positively charged at higher pH values. The pH of zero charge of the FeX7502h material was somehow intermediate between the other two values. In a previous work [38], however, it was shown that the negative surface charge of these magnetic adsorbents did not hamper interaction with (negatively charged) DNA moieties, since, besides electrostatic interactions, van der Waals forces and especially H-bonds also occurred. Such processes are expected to be even more complex when DNA separation occurs in human blood. 

As a whole, the reported physico-chemical characterization showed that the obtained materials were mainly amorphous and had a more heterogeneous surface with respect to core–shell particles with a silica shell (like Chemicell NPs, for instance). Besides amorphous silica, the resulting matrix embedded the Fe^0^ NPs and contained also aluminum oxides/hydroxides (deriving from the zeolite framework collapse) and other Fe-containing phases (FeOx and, in two samples, Fe_2_SiO_4_, as detailed in Table 2). 

**Figure 4:**
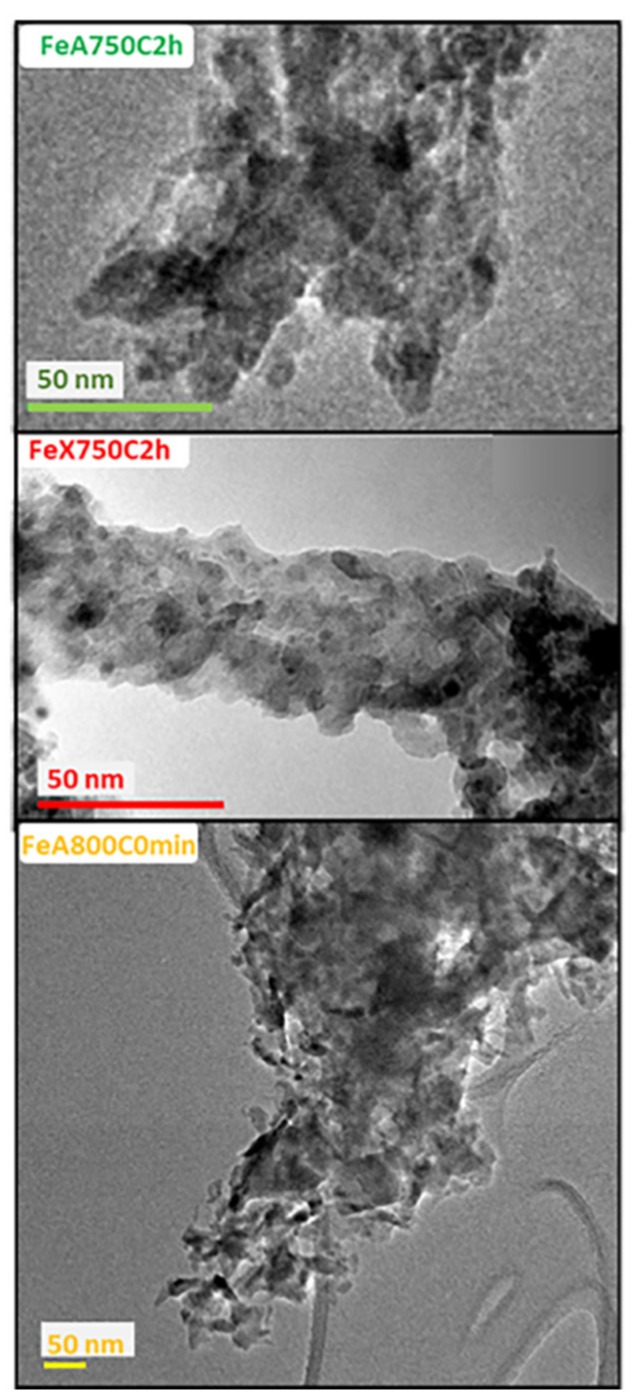
Selected TEM micrographs of the FeX750C2h, FeA750C2h, and FeA800C0min magnetic adsorbents.

Figure 4 reports the TEM images of the FeX750C2h, FeA750C2h, and FeA800C0min magnetic adsorbents. With all the three samples, Fe^0^ nanoparticles having different sizes were homogeneously dispersed in a prevailingly amorphous matrix. In previous works [37,38], both the particle size distribution and the average particle size were determined by TEM analysis and here, for the sake of brevity, only the most relevant results are mentioned. In particular, the Fe^0^ nanoparticles’ average diameter was 4.4, 6.2, and 6.0 nm in FeX750C2h, FeA750C2h, and FeA800C0min, respectively [37,38]. It must be said that also few larger Fe^0^ particles, with dimension in the 50–150 nm range, were detected in all the three samples (not reported), but their number was so small that it did not sensibly alter the particle size distribution [37,38]. The slightly smaller particle size observed with the FeX750C2h sample, as compared to the two samples obtained from the Fe-A zeolite, could be due to the parent zeolite structure, since cation sites in the X zeolite (Si/Al = 2.5) were more diluted (far from each other) than in the A zeolite, which had a lower Si/Al ratio (Si/Al = 1.0), and, thus, smaller Fe^0^ nanoparticles were obtained. Further considerations on the actual relevance of the difference particle size can be drawn by the magnetization measurements reported in the following paragraph.

### 2.2. Magnetic Characterization

**Figure 5:**
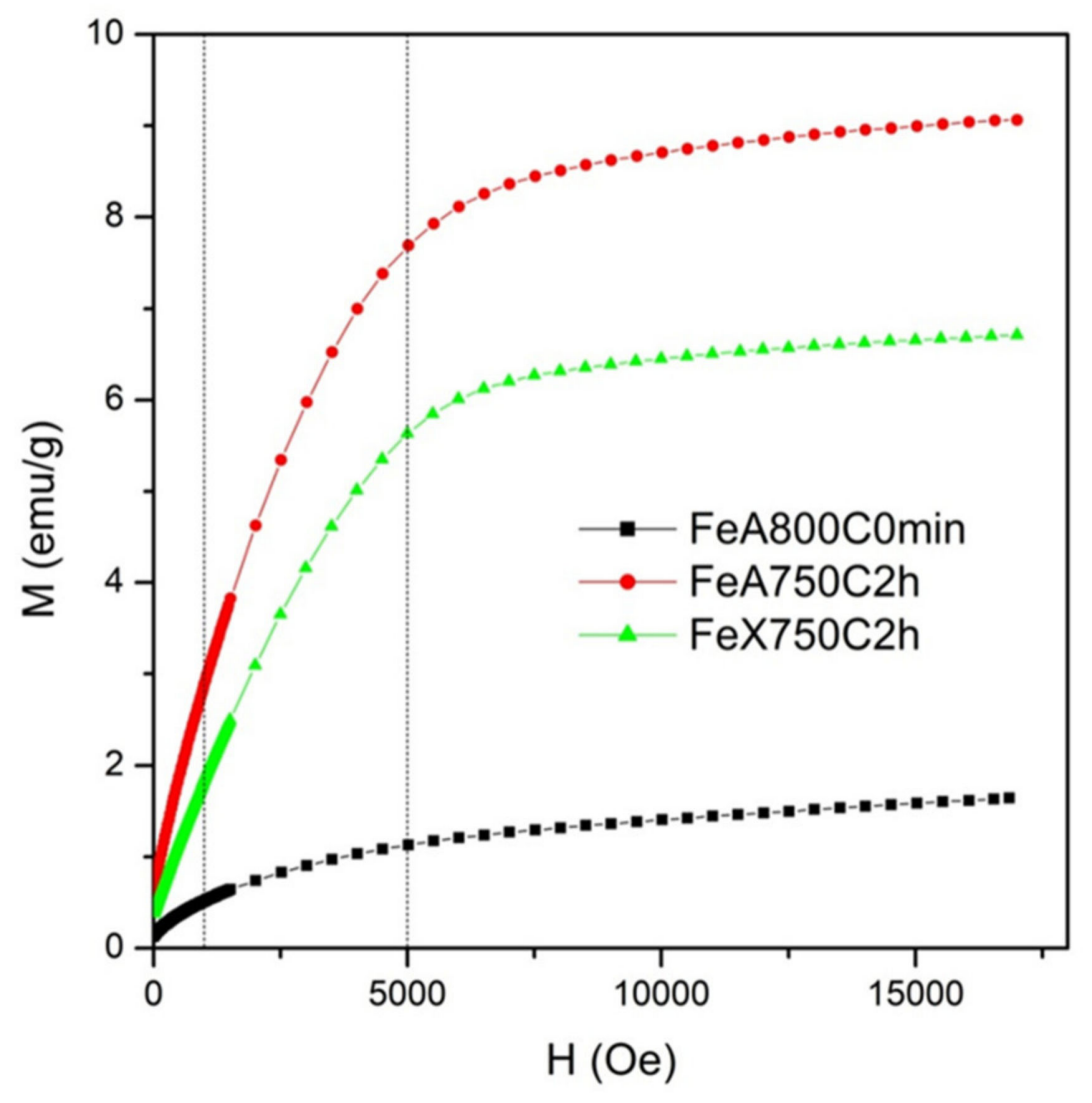
Room-temperature magnetization curves in the first quadrant for the three examined samples.

The magnetic properties of the three adsorbents were magnetically characterized at room temperature using a vibrating sample magnetometer in the range 0–1.7 × 10^4^ Oe. The equilibrium magnetization curves in the first quadrant are reported in Figure 5. 

All the examined samples were characterized by the presence of a saturating magnetic component followed by a linear increase of the magnetization curve at high fields. The magnetic signal turned out to be considerably lower in the FeA800C-0 sample than in the other two samples. These results were compatible with the co-presence of magnetic Fe^0^ nanoparticles and of a fraction of transition-metal cations still dispersed in the samples; the present results indicated that—in the examined samples—the total number of magnetic nanoparticles was higher when the annealing temperature was lower and the reduction time higher, and that a role was also played by the type of starting zeolite. Although it is not possible to safely infer the effect of annealing temperature and time on the number of magnetic nanoparticles from just two cases, it is reasonable to assume that the observed behavior reflects a more general rule, considering that both nucleation and kinetics of the reduction reaction become secondary effects with increasing the temperature of annealing, being overcome at higher temperature by the process of coalescence among particles (acting to produce less particles of larger size) and by the increase of reactivity between metal particles and the matrix (acting to produce, above about 750 °C, iron-rich silicate or iron-rich spinel phases, characterized by a room-temperature magnetic response entirely different from that of metallic nanoparticles). 

The magnetic force on a single micrometric grain of annealed material containing a significant number of Fe^0^ nanoparticles was evaluated for each of the three samples considering the overall grain magnetic moment as resulting from the magnetization of the embedded Fe^0^ nanoparticles. The force was evaluated under an applied magnetic field of either 1 or 5 kOe (0.1/0.5 T, vertical dashed lines in Figure 5) and a magnetic-field gradient of either 1 kOe/cm or 5 kOe/cm (=10/50 T/m, compatible with the behavior in space of the stray field of a commercial NdFeB bar magnet). For these values of the applied field, the paramagnetic contribution of magnetic ions was negligible.

Using the pairs of lowest/highest values of B and ▽B, the following limiting values of the magnetic force F_m_ on a single magnetic grain were obtained: for an average diameter of 7.5 mm (typical of grains existing in the two annealed samples obtained from A zeolite) [36], F_m,min_ = 2.8 × 10^−12^ N; F_m,MAX_ = 3.1 × 10^−11^ N (Fe800C0min) and F_m,min_ = 1.6 × 10^−11^ N; F_m,MAX_ = 2.1 × 10^−10^ N (sample FeA750C2h); for an average grain diameter of 3.8 mm (typical of the sample obtained from X zeolite) [36], F_m,min_ = 1.3 × 10^−12^ N; F_m,MAX_ = 2.0 × 10^−11^ N (FeX750C2h). Intermediate values of force were obtained for intermediate values of both B and ▽B.

The reported values were all well above the typical value of the random forces of thermal origin acting on the grain at room temperature [42,43], so that the deterministic path of the grain by the effect of the magnetic field gradient was not disrupted by random thermal fluctuations. 

On the other hand, if one assumes a drift velocity of magnetic grains of the order of 1.67 × 10^−4^ m s^−1^ (1 cm per minute, appropriate to magnetophoresis of nanoparticle aggregates or large grains [44]) the viscous force in a fluid with viscosity comparable to water turned out to be of the order of 1.1 × 10^−11^ N in samples Fe800C0min and FeA750C2h, whereas it was lower (of the order of 5.3 × 10^−12^ N) for FeX750C2h. 

As a consequence, all the examined samples can be effectively exploited for steady state magnetophoresis and/or magnetically supported separation in a fluid environment, although the use of a strong magnet is recommended in the case of the sample exhibiting the lowest magnetic signal. 

### 2.3. Separation of Biological Entities 

Table 3 (Materials and Methods) summarizes the performed biological separations; a careful examination of the reported results allowed us to infer some considerations concerning several related practical, technological, and scientific aspects. 

As a whole, all the tested adsorbents (including Chemicell NPs) were very effective towards the separation of biological entities in actual operative conditions (i.e., those of a molecular diagnostic laboratory). In many cases, the obtained C_t_ parameter values (the lower its value, the higher the amount of biological entity initially separated by the adsorbent and, thus, the more effective the adsorbent itself, as detailed in the Materials and Methods section) were comparable or better than those obtained with the benchmark system (Chemicell NPs). This observation confirms the actual possibility of using the proposed magnetic adsorbents for biological separation. 

Concerning the crucial role of the adopted protocol, as mentioned in the Material and Methods section, Protocol 1 was set up with the purpose of obtaining the best biological separations by using Chemicell NPs. Indeed, according to the usual classification of magnetic adsorbents [38], Chemicell NP is a type 2 system (i.e., consisting of nanoparticles with a magnetic core coated by a shell to which biological entities bind), whereas the proposed magnetic adsorbents are type 3 materials (i.e., consisting of magnetic nanoparticles dispersed within a heterogeneous and still porous matrix, to which biological entities bind). Thus, to optimize the performance of so markedly different magnetic adsorbents, different protocols are required. 

The last consideration fairly explains the results of Separations 1, 2, and 3 concerning the gene target RNASE; when Protocol 1 was used, the here proposed magnetic adsorbent’s performance was worse (with respect to Chemicell NPs), whereas it increased (i.e., lower C_t_ values were obtained) when Protocols 4 and 5 were adopted, i.e., when both i) the adsorbents were dehydrated at a higher temperature and ii) the elution step was performed at higher temperature than in Protocol 1. Similarly, with the gene target factor V, better performances of the magnetic adsorbents were obtained by adopting Protocols 4 and 5 (implying higher temperature of both dehydration and elution than Protocols 1, 2, and 3). The positive effect of both dehydration and elution temperature is likely related to the material surface properties; indeed, the here proposed magnetic adsorbents were characterized by a plethora of surface species (amorphous silica and alumina phases, Fe-containing phases, etc.) and, in the case of FeA800C0min, also residual microporosity due to incomplete zeolite collapse. The aforementioned surface species were very heterogeneous in nature and may require higher temperature to remove adsorbed water. Only by this way, surface active sites can become available for the interaction with DNA moieties. Identifying the precise type of DNA-surface interaction is not straightforward, especially in the present case. Indeed, several authors [5,45,46] found that DNA adsorption on silica (a much simpler material) is related to i) shielded intermolecular electrostatic forces; ii) dehydration of DNA and silica surface; and iii) intermolecular H bond formation in the DNA-silica contact layer. Those are likely the types of interactions occurring at the surface of Chemicell NPs, which have a silica shell, simultaneously acting as adsorbing surface and as shield of the Fe-containing core, which has “only” a magnetic (i.e., not adsorbent) role. Whilst the Fe-containing phase in Chemicell NPs is not accessible to target biomolecules, the here proposed adsorbents had a much more heterogeneous (i.e., containing different surface species) and porous exposed surface, which also had macropores that were accessible to DNA moieties, as shown by confocal microscopy technique in a previous work [38]. Besides the functional groups of hydrated amorphous silica, other different types of adsorbing sites were likely accessible to target biomolecules, with which DNA moieties could interact through stronger interactions than mere dispersion forces, implying not only H bonding, but also interactions with other polar/ionic species (likely, surface uncoordinated Al^3+^ and Fe^3+^ ions). These species can strongly interact with DNA moieties that contain both N atoms and phosphate groups, both able to chelate iron species, and, therefore, require optimized protocols implying for instance elution at a higher temperature. On the basis of the previous considerations, however, a thorough comprehension of the complex interplay occurring between DNA moieties and the surface of the proposed magnetic adsorbents seems a rather complicated task, so far, given the data available to us. Blood itself is a very complex matrix, where iron species in hemoglobin may inhibit DNA polymerase. Actually, other experiments and ad hoc investigations are likely required to unravel the type of DNA-surface interactions taking place in the studied systems, which could be the topic of a forthcoming study.

To the best of our knowledge, the most important results were obtained in Separation 9, during which different amounts of human blood (0.1, 1.0, 10.0, 100.0 µL) were tested. Apparently, the lower the amount of blood used, the higher the C_t_ value obtained, but at low blood volume (namely, 0.1, 1.0, and 10.0 µL), the proposed magnetic adsorbents performed better than Chemicell NPs, whereas the contrary occurred by using 100.0 µL of blood. This finding points out the high sensitivity of the proposed adsorbents, suggesting their application for analysis with extremely low amounts of DNA, i.e., when a tiny amount of blood was available for the separation.

As far as the Separations 10 and 10bis are considered, it must be pointed out that the bacterial DNA (100 CFU/mL) was successfully separated both in manual and automated mode, indicating that the proposed adsorbents could be used in actual separations, due to their magnetic properties. On the other hand, higher C_t_ values were obtained with the FeA800C0min sample, notwithstanding the (optimized) Protocol 4 was adopted. In this respect, it must be pointed out that, for instance, the bacterial genome is much smaller than the human one (ca. three orders of magnitude) and that the obtained results could be implemented by finding a proper separation protocol. 

A comparison of the overall performance of the three magnetic adsorbents is not straightforward, although some considerations are possible, looking, for instance, at the separations carried out by following the (optimized) Protocols 4 and 5; the FeX750C2h and FeA750C2h adsorbents had very similar behaviors (i.e., close values of C_t_), whereas (with exception of the *Staphylococcus aureus* separations) the FeA800C0min adsorbent had normally a better performance (i.e., lower C_t_ values) than the FeA750C2h adsorbent, likely due to its higher specific surface area and higher point of zero charge, both properties favoring interaction with (negatively charged) DNA moieties. From this point of view, the results obtained with the FeA800C0min adsorbent during the *Staphylococcus aureus* DNA separations (i.e., higher C_t_ values) could be due to a facile interaction with (smaller) DNA moieties, which, being strongly adsorbed, could not be eluted, with a consequent increase of the corresponding C_t_ values.

Finally, from a practical point of view, the use of the proposed magnetic adsorbents in both manual and automated mode (to the same extent of Chemicell NPs) is particularly sound, indicating that the proposed materials have adsorption and magnetic properties allowing automated procedures, which are highly desirable, especially in actual laboratories analysis. 

## 3. Material and Methods

### 3.1. Chemicals and Materials Synthesis

**Scheme 1:**
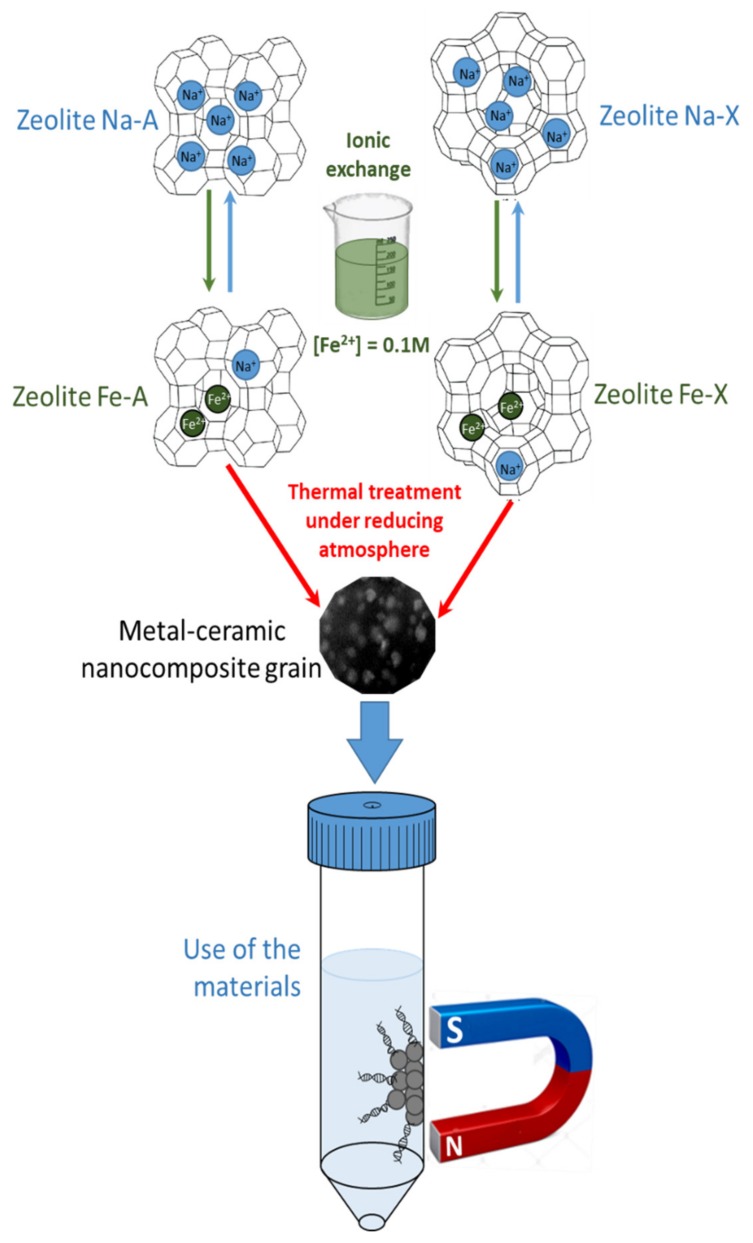
Scheme (not in scale) of the preparation procedure of the magnetic adsorbents by Fe^2+^ exchange of either Na-A or Na-X zeolite and successive reducing thermal treatment.

Carlo Erba (city, Italy) reagent grade 4A and 13X zeolites (sodium forms, hereafter labeled as Na-A and Na-X zeolite, respectively), the respective framework type and chemical formula being LTA-Na_12_Al_12_Si_12_O_48_^.^27H_2_O and FAU-Na_86_Al_86_Si_106_O_384_^.^264H_2_O [47]. The cation exchange capacity of both zeolites was experimentally determined by following recommendations of the “batch exchange method” [48,49]. The obtained values were very close to the calculated cation exchange capacities, i.e., 5.48 (Na-A) and 4.73 meq g^−1^ (Na-X). The preparation procedure of the magnetic adsorbents is depicted in Scheme 1. 

In a typical exchange procedure, a proper amount of Na-zeolite was contacted with a 0.1 M Fe^2+^ solution, prepared by using 99.5 wt.% FeSO_4_⋅7H_2_O (Aldrich) in ddH_2_O (double-distilled water; ion exchange conditions: solid/liquid weight ratio = 1/50 g/g; contact time *t* = 40 min). Fe^2+^ exchange was performed at about 7 °C under Ar to prevent Fe^2+^ oxidation, according to [50]. The solid was, then, separated from the liquid by filtration and contacted again with a fresh solution. In total, the procedure was iterated 8 and 5 times with Na-A and Na-X zeolite, respectively, due to their different exchange capacities [51,52]. The Fe^2+^ exchanged zeolites (hereafter referred to as Fe-A and Fe-X zeolite) were washed with ddH_2_O, dried at 80 °C for 24 h, and stored for at least 72 h in a 50% relative humidity environment (as obtained by using a saturated Ca(NO_3_)_2_ solution); this storage method resulting in the zeolite water saturation. Then, both the Fe-A and Fe-X zeolite were subjected to various thermal treatments under reducing atmosphere, obtained by putting the powder in a Pt crucible inside an Al_2_O_3_ tubular furnace (inner diameter = 6.9 cm, height = 91 cm) and flowing a 2 vol.% H_2_ in Ar mixture. In order to obtain the FeA750C2h and FeX750C2h samples (Table 1), each Fe-exchanged zeolite was heated up to the target temperature (temperature ramp: 10° min^−1^), kept at this temperature for the fixed time, and then the furnace was switched off and the sample was left to cool down to room temperature. In order to obtain the FeA800C0min sample (Table 1), the Fe-A zeolite was heated up to the fixed temperature (temperature ramp: 10° min^–1^), and as soon as the temperature was reached, the furnace was switched off and the sample was left to cool down to room temperature. 

### 3.2. Characterization Methods

The contents of both Fe^2+^ and residual Na^+^ ions in the exchanged zeolites were determined by atomic absorption spectrophotometry (AAS, Perkin-Elmer AAnalyst 100 apparatus, Milano, Italy) after dissolving the solids in a 40 wt.% HF and 14 wt.% HClO_4_ aqueous solution [53,54]. 

The X-ray powder diffraction (XRPD) characterization (along with Rietveld quantitative phase analysis), the calculation of the specific surface area according to the Brunauer–Emmett–Teller (BET) method (S_BET_), the determination of total pore volume (V_p_) and micropore volume (V_mp_), the TEM (transmission electron microscopy) characterization, and the magnetic characterization of the three magnetic adsorbents are detailed in [37,38]. 

The ζ-potential curves in deionized water were obtained by measuring the electrophoretic mobility as a function of pH at 25 °C by means of electrophoretic light scattering (ELS) (Zetasizer Nano-ZS, ZEN5600, Malvern Instruments, Worcestershire, UK). Suspensions were obtained after 2 min of sonication with an ultrasonic probe (100 W, 20 kHz, Sonoplus; Bandelin, Berlin, Germany), and pH was adjusted by adding either 0.1 M HCl or 0.1 M NaOH [55]. 

### 3.3. Separation of Biological Entities

Volunteers’ blood samples (from Nurex) were tested; for *Staphylococcus aureus* separation, bacteria contamination was performed post-sampling.

Separation of the target gene factors V and RNASE and of the bacterium *Staphylococcus aureus* from human blood were performed by using the FeA750C2h, FeA800C0min, and FeX750C0min magnetic adsorbents and one of the best performing commercial separation system, namely SiMAG-N-DNA particles, produced by Chemicell, referred to as Chemicell NPs (N-DNA particles). According to the producer, Chemicell NPs consist of ca. 1μ particles of superparamagnetic maghemite, coated with a porous silica shell (obtained by precipitation method), with a surface area of ca. 100 m^2^ g^−1^ that are commercialized as vials containing 1 mL of 100 mg/mL aqueous suspension.

The amount of the biological entity separated from the human blood was evaluated by real time PCR [56,57]. In this method, the amount of DNA originally obtained in the separation is amplified many times by iterating 30–40 times the following steps:

1. DNA denaturation, during which the two filaments are separated (at temperature > 90 °C);
2. Duplication of the separated DNA filaments by proper nucleotides and action of the polymerase enzyme (at about 50–60 °C);
3. Extension of the DNA chain by enzyme polymerase (at about 70–75 °C).

Such iteration gives rise to an exponential growth of the amount of the originally separated DNA. The results of such exponential growth are presented in an amplification curve, which reports the intensity of the fluorescent signal of a component bound to DNA as a function of the number of cycles of iteration. The amplification curve starts from a baseline, then grows exponentially and, finally, attains a plateau. From the curve, the value of the “threshold cycle” (C_t_) may be detected. Such value represents the first cycle of iteration in which the fluorescent signal of the component, bound to DNA, becomes evident. Thus, the lower the C_t_ value recorded in the process, the higher the amount of DNA originally separated by the adsorbent used, and the better its performance in the separation.

In this work, the biological entities’ separations were performed according to the six protocols detailed below.

*Protocol 1*: This protocol, which is a modification of the method described by Boom et al. [3], proved to be particularly suitable in separations made by using Chemicell NP; 1 mL of chaotropic salt guanidinium isothiocyanate solution was added to 25 µL Chemicell NP suspension (or one of our magnetic adsorbents). The obtained suspension was mixed to 50 µL of human blood and kept under continuous stirring for 10 min.

In this lag of time, the chaotropic salt gives rise to cell lysis and favors interaction between the biological entity and the surface of the adsorbent. Then, Chemicell NP (or another magnetic adsorbent) were separated from the solution, by using an external magnet, washed twice with a 70 wt.% ethanol in water solution, washed once with acetone, and finally dried at 60 °C for 10 min (these steps allowed removal of undesired organic substances). The biological entities adsorbed on the surface of Chemicell NP (or other magnetic adsorbent) were eluted by adding water at 60 °C and keeping it under continuous stirring for 10 min. The yield of the biological separation was evaluated by real time PCR, reporting the value of C_t_.

As Protocol 1 did not prove to be proper for the magnetic adsorbents under study, the following other protocols were set up.

*Protocol 2:* 25 µL of a suspension formed by Chemicell NP (or one of the magnetic adsorbents studied here) water and guanidinium isothiocyanate (prepared as in Protocol 1) was added to 50 µL of human blood and kept under continuous stirring for 10 min. Then, Chemicell NPs (or of one of the other magnetic adsorbents) were separated from the solution by using an external magnet, washed twice with a 70 wt.% ethanol in water solution, and washed once with acetone (to remove undesired organic substances). The biological entities adsorbed on the surface of Chemicell NPs (or on one of the other magnetic adsorbent) were eluted by adding water at 60 °C and continuously stirring for 10 min. The yield of the biological separation was evaluated by real time PCR, reporting the value of C_t_.

*Protocol 3*: 300 mg magnetic adsorbent was suspended in water (from 120 to 150 mg/mL), and then 1 mL of guanidinium isothiocyanate was added. Afterwards, 50 µL of human blood was added to the suspension and kept under continuous stirring for 10 min. Then, the magnetic adsorbent was separated from the solution by using an external magnet, washed twice with a 70 wt.% ethanol in water solution, washed once with acetone, and finally dried at 60 °C for 10 min (to remove undesired organic substances). The biological entities adsorbed on the surface of the magnetic adsorbent were eluted by adding water at 60 °C and continuously stirring for 10 min. The yield of the biological separation was evaluated by real time PCR, reporting the value of C_t_.

*Protocol 4*: 300 mg magnetic adsorbent was suspended in water (from 120 to 150 mg/mL) and then 1 mL of guanidinium isothiocyanate was added. Afterwards, 50 µL of human blood was added to the suspension and kept under continuous stirring for 10 min. Then, the magnetic adsorbent was separated from the solution by using an external magnet, washed twice with a 70 wt.% ethanol in water solution, washed once with methanol, and finally dried at 85 °C for 10 min (to remove undesired organic substances). The biological entities adsorbed on the surface of the magnetic adsorbent were eluted by adding water at 80 °C and continuously stirring for 10 min. The yield of the biological separation was evaluated by real time PCR, reporting the value of C_t_.

*Protocol 5*: 300 mg magnetic adsorbent was suspended in water (from 120 to 150 mg/mL) and then 1 mL of guanidinium isothiocyanate was added. Afterwards, 50 µL of human blood was added to the suspension and kept under continuous stirring for 10 min. Then, the magnetic adsorbent was separated from the solution by using an external magnet, washed twice with a 70 wt.% ethanol in water solution, and washed once with ddH_2_O. The biological entities adsorbed on the surface of the magnetic adsorbent were eluted by adding water at 80 °C and continuously stirred for 10 min. The yield of the biological separation was evaluated by real time PCR, reporting the value of C_t_.

*Protocol 6*: 948 mg magnetic adsorbent was added to 7.5 mL of a solution of both guanidinium isothiocyanate (8 M) and Na_3_N (0.01 wt.%) in water. Afterwards, 50 µL of human blood was added to the suspension and kept under continuous stirring for 10 min. Then, the magnetic adsorbent was separated from the solution by using an external magnet, washed twice with a 70 wt.% ethanol in water solution, washed once with methanol, and finally dried at 85 °C for 10 min (to remove undesired organic substances). The biological entities adsorbed on the surface of the magnetic adsorbent were eluted by adding water at 80 °C and continuously stirring for 10 min. The yield of the biological separation was evaluated by real time PCR, reporting the value of C_t_.

All the details of the performed biological separations with indication of separated biological entity, used magnetic adsorbent, followed protocol, adopted modality, and obtained results (reported as “threshold cycle”, C_t_) are listed in Table 3. Some biological separations were performed manually by an operator, whereas some other were performed by adopting an automated procedure implying the use of a robot, as detailed in Table 3. Moreover, some changes to the above protocols were adopted to investigate the role of the amount of blood used in the biological separations (Separation 9, Table 3), and in some cases the separation runs were performed more than once (Separation 4, Table 3). 

## 4. Conclusions

A facile two step procedure, based on the ionic exchange of (low cost) commercial zeolites, followed by thermal treatment under reducing atmosphere, allowed for the production of three magnetic metal–ceramic nanocomposites that performed as very efficient adsorbents during some biological separations in real conditions, as those normally encountered in molecular diagnostics laboratories. 

With respect to other available systems for biological separations, a point of novelty here lays in the process by which the magnetic adsorbents are produced. Actually, commercial systems are obtained by powerful (but sophisticated) synthetic techniques (such as the sol–gel method) that require expensive precursors, skilled chemists, and multi-step procedures. The magnetic adsorbents proposed here are, instead, obtained starting from low-cost precursors (i.e., commercial zeolite) by a simple two-step procedure, i.e., a kind of process that could be scaled up, reducing costs of the adsorbent materials, by using, for instance, a fluidized bed reactor. With respect to the laboratory furnace used in this work, such type of reactor could indeed allow a more efficient and intimate contact between the Fe-exchanged zeolite particles and the reducing gas, leading to a further implementation of the proposed materials. Moreover, the use of a fluidized bed reactor allows dozens of kilograms of final product to be produced per batch of reaction, which together with the low cost of raw materials, would result in an overall low-cost process. Since so far no optimization of the process has been performed, there is still room for further improvement to the features of the obtained magnetic adsorbents and, likely, also their performance towards biological separations. 

In particular, among the various keys that are available to tune the process of production of the magnetic adsorbents, zeolites with a slightly higher Si/Al ratio could be used as raw materials, allowing us to obtain magnetic adsorbents more similar to silica, a material that allows very good results in biological separations to be attained. As a whole, however, the materials studied in this paper showed separation performances mostly comparable and sometimes better than that of the commercial system adopted as a benchmark, showing very good sensitivity in tests carried out with extremely small blood volumes. Indeed, blood is a difficult-to-treat matrix, due to the presence of iron sites in hemoglobin, inhibiting DNA polymerase. The here obtained results point out the fact that the system proposed here, i.e., the magnetic adsorbents along with optimized protocols for real-time PCR detection, allows extremely high selectivity finally overcoming the issues of tiny blood volumes and presence of hemoglobin. 

Finally, due to their magnetic behavior, the proposed adsorbents could be employed in both manual and automatized processes, the latter ones being highly desirable in molecular diagnostic laboratories, where a large number of analysis have to be carried out, especially in critical conditions, like during epidemic episodes, for instance.

## Figures and Tables

**Table 1:** Parent Fe-exchanged zeolite, temperature, length of the reduction treatment, and name of the obtained sample.

Zeolite	Temperature	Length	Sample
Fe-A	750 °C	2 h	FeA750C2h
Fe-A	800 °C	0 min	FeA800C0min
Fe-X	750 °C	2 h	FeX750C2h

**Table 2:** Results of QPA as obtained by Rietveld refinement (phase wt.%). Specific surface area (S_BET_); total pore volume (V_p_); micropore volume (V_mp_), as obtained by N_2_ isotherms at –196 °C.

Magnetic Adsorbent	Parent Zeolite (wt.%)	Fe^0^ (wt.%)	Fe_2_SiO_4_ (wt.%)	Amorphous Phase (wt.%)	S_BET_ (m^2^ g^−1^)	V_p_ (cm^3^ g^−1^)	V_mp_ (cm^3^ g^−1^)
FeA800C0min	2.4	0.3	11.5	85.8	152	0.42	0.051
FeA750C2h	1.0	2.6	7.0	89.4	9.50	0.055	0.007
FeX750C2h	-	1.6	-	98.4	19.03	0.066	0.029

**Table 3:** Summary of the performed biological separations and obtained results.

**SEPARATION 1**									
**Biological Entity**	**Adsorbent**	**Protocol**	**Modality**	**C_t_**					
Gene target RNASE	FeA800C0min	1	manual	30					
Gene target RNASE	FeA750C2h	1	manual	32					
Gene target RNASE	Chemicell NP	1	manual	25					
**SEPARATION 2**									
**Biological Entity**	**Adsorbent**	**Protocol**	**Modality**	**C_t_**					
Gene target RNASE	FeA750C2h	4	manual	24					
Gene target RNASE	FeX750C2h	4	manual	23.4					
Gene target RNASE	Chemicell NP	1	manual	24.7					
**SEPARATION 3**									
**Biological Entity**	**Adsorbent**	**Protocol**	**Modality**	**C_t_**					
Gene target RNASE	FeA750C2h	5	manual	23.5					
Gene target RNASE	FeX750C2h	5	manual	23					
Gene target RNASE	Chemicell NP	1	manual	24.7					
**SEPARATION 4** (repeated 3 times)									
**Biological Entity**	**Adsorbent**	**Protocol**	**Modality**	**C_t_**					
Gene target factor V	FeA800C0min	1	manual	26		27		28	
Gene target factor V	FeA750C2h	1	manual	33		27.8		31.5	
Gene target factor V	Chemicell NP	1	manual	26		26		27.4	
**SEPARATION 5**									
**Biological Entity**	**Adsorbent**	**Protocol**	**Modality**	**C_t_**					
Gene target factor V	FeA800C0min	2	manual	27.7					
Gene target factor V	Chemicell NP	2	manual	26.7					
**SEPARATION 6**									
**Biological Entity**	**Adsorbent**	**Protocol**	**Modality**	**C_t_**					
Gene target factor V	FeA800C0min	3	manual	27					
Gene target factor V	FeA750C2h	3	manual	28					
Gene target factor V	Chemicell NP	3	manual	24.9					
**SEPARATION 7** (repeated 3 times, average value reported)									
**Biological Entity**	**Adsorbent**	**Protocol**	**Modality**	**C_t_**					
Gene target factor V	FeA800C0min	4	manual	25.6					
Gene target factor V	FeA750C2h	4	manual	25.4					
Gene target factor V	Chemicell NP	4	manual	26					
**SEPARATION 7bis** (repeated 3 times, average value reported)									
**Biological Entity**	**Adsorbent**	**Protocol**	**Modality**	**C_t_**					
Gene target factor V	FeA800C0min	4	manual	23.6					
Gene target factor V	FeA750C2h	4	manual	24.7					
Gene target factor V	Chemicell NP	4	manual	24					
**SEPARATION 8** (repeated 3 times, average value reported)									
**Biological Entity**	**Adsorbent**	**Protocol**	**Modality**	**C_t_**					
Gene target factor V	FeA800C0min	5	manual	27.3					
Gene target factor V	FeA750C2h	5	manual	28.5					
Gene target factor V	Chemicell NP	5	manual	25.6					
**SEPARATION 8bis** (repeated 3 times, average value reported)									
**Biological Entity**	**Adsorbent**	**Protocol**	**Modality**	**C_t_**					
Gene target factor V	FeA800C0min	5	manual	24.6					
Gene target factor V	FeA750C2h	5	manual	23.3					
Gene target factor V	Chemicell NP	5	manual	24					
**SEPARATION 9** (performed adding different amounts of blood)									
**Biological Entity**	**Adsorbent**	**Protocol**	**Modality**	**C_t_ at Blood Volume (µL)**					
				100.0	10.0		1.0		0.1
Gene target factor V	FeA800C0min	4	manual	25.7	28		30.8		34
Gene target factor V	FeA750C2h	4	manual	26	26.3		30		35
Gene target factor V	Chemicell NP	4	manual	24.1	28.4		32		35.7
**SEPARATION 10** (repeated 3 times, average value reported)									
**Biological Entity**	**Adsorbent**	**Protocol**	**Modality**	**C_t_**					
*Staphylococcus aureus*	FeA800C0min	4	manual	35					
*Staphylococcus aureus*	FeA750C2h	4	manual	33.4					
*Staphylococcus aureus*	Chemicell NP	4	manual	32.4					
**SEPARATION 10 bis** (repeated 3 times, average value reported)									
**Biological Entity**	**Adsorbent**	**Protocol**	**Modality**	**C_t_**					
*Staphylococcus aureus*	FeA800C0min	4	automated	35.2					
*Staphylococcus aureus*	FeA750C2h	4	automated	33.3					
*Staphylococcus aureus*	Chemicell NP	4	automated	33.5					
